# CD36 promotes the epithelial–mesenchymal transition and metastasis in cervical cancer by interacting with TGF-β

**DOI:** 10.1186/s12967-019-2098-6

**Published:** 2019-10-26

**Authors:** Min Deng, Xiaodong Cai, Ling Long, Linying Xie, Hongmei Ma, Youjian Zhou, Shuguang Liu, Chao Zeng

**Affiliations:** 1 Department of Pathology, The Eighth Affiliated Hospital, Sun Yat-sen University, Futian District, Shenzhen, 518033 Guangdong China; 2 Affliated Cancer Hospital & Institute of Guangzhou Medical University, Guangzhou, 510095 Guangdong China; 3 Department of Pathology, Guangdong Medical University, Dongguan, 523808 Guangdong China; 4 Department of Neurology, The Sixth Affiliated Hospital of Sun Yat-Sen University, Guangzhou, 510655 Guangdong China; 5 Department of Neurology, Third Affiliated Hospital of Sun Yat-sen University, Guangzhou, 510600 Guangdong China

**Keywords:** CD36, Cervical cancer, EMT, TGF-β, Metastasis

## Abstract

**Background:**

Accumulating evidence indicates that CD36 initiates metastasis and correlates with an unfavorable prognosis in cancers. However, there are few reports regarding the roles of CD36 in initiation and metastasis of cervical cancer.

**Methods:**

Using immunohistochemistry, we analyzed 133 cervical cancer samples for CD36 protein expression levels, and then investigated the correlation between changes in its expression and clinicopathologic parameters. The effect of CD36 expression on the epithelial–mesenchymal transition (EMT) in cervical cancer cells was evaluated by Western immunoblotting analysis. In vitro invasion and in vivo metastasis assays were also used to evaluate the role of CD36 in cervical cancer metastasis.

**Results:**

In the present study, we confirmed that CD36 was highly expressed in cervical cancer samples relative to normal cervical tissues. Moreover, overexpression of CD36 promoted invasiveness and metastasis of cervical cancer cells in vitro and in vivo, while CD36 knockdown suppressed proliferation, migration, and invasiveness. We demonstrated that TGF-β treatment attenuated E-cadherin expression and enhanced the expression levels of CD36, vimentin, slug, snail, and twist in si-SiHa, si-HeLa, and C33a–CD36 cells, suggesting that TGF-β synergized with CD36 on EMT via active CD36 expression. We also observed that the expression levels of TGF-β in si-SiHa cells and si-HeLa cells were down-regulated, whereas the expression levels of TGF-β were up-regulated in C33a–CD36 cells. These results imply that CD36 and TGF-β interact with each other to promote the EMT in cervical cancer.

**Conclusions:**

Our findings suggest that CD36 is likely to be an effective target for guiding individualized clinical therapy of cervical cancer.

## Background

Cervical cancer is regarded as a common malignancy causing cancer-related deaths among women worldwide [1]. The initiation and progression of cervical cancer is a multi-step process that entails high-risk human papillomavirus (HPV) infection, cervical intraepithelial neoplasia [2], epithelial–mesenchymal transition (EMT), metastasis, and invasion. The majority of early cancer is now detected through refined detection technology, such as cervical cytology and biopsy, and the incidence of cervical cancer has notably diminished [3]. However, patients with advanced cervical cancer still have unfavorable outcomes due to the high incidence of metastasis, which is one of the main factors influencing the prognosis of patients. Thus, it is of great importance to study the underlying mechanism regarding the metastasis of cervical cancer.

CD36, also known as a scavenger receptor, is a 88-kd transmembrane glycoprotein that is expressed on macrophages [4, 5], platelets, monocytes, endothelial cells, and adipocytes [6]. CD36 regulates various biologic functions under physiologic or pathologic conditions, including angiogenesis and atherosclerosis. CD36 also uses multiple receptors, such as collagen type I receptor, thrombospondin-1 (TSP-1) receptor [7], endothelial receptor, and fatty acid receptor [8–10]. Moreover, CD36 participates in the clearance of cellular apoptosis, macrophage phagocytosis, inflammation, and fatty acid metabolism [10–12]. An increasing number of studies have found that CD36 plays a vital role in the development of cancers, especially as it relates to the process of cancer metastasis. The presence of CD36 on cancer cells initiates metastasis and correlates with an unfavorable prognosis for melanoma and breast cancer, and inhibition of CD36 impairs metastasis [13]. There are currently few reports on the expression and actions of CD36 in cervical cancer. Thus, investigation of a new molecular mechanism for CD36 in cervical cancer metastasis is critical in improving patient prognosis.

EMT is a complicated process in which epithelial cells acquire a mesenchymal phenotype, directly contributing to invasion and metastasis of the malignancy [14]. CD36 activates the Wnt/β-catenin signaling pathway to drive EMT in hepatoma cells when combined with FFA [15]. Furthermore, inhibition of CD36 expression activates the smad2 and ERK1/2 pathways by regulating the expression of TGF-β (transforming growth factor-β), thus preventing the expression of fibronectin and ultimately alleviating the occurrence of EMT in renal tubular epithelial cells [16]. In the present study, we aimed to demonstrate that CD36 acts as a novel carcinogenic factor in TGF-β-mediated EMT in cervical cancer.

## Materials and methods

### Reagents and antibodies

Streptavidin-perosidase (SP) and diaminobenzidine (DAB) kits were purchased from Maixin Biotechnology Company (Fuzhou, China). The following primary antibodies were used for Western blot analysis: anti-CD36 (Santa Cruz, CA, USA), anti-E-cadherin, anti-TGF-β, anti-vimentin, anti-snail, anti-slug, anti-twist (Proteintech, IL, USA), and anti-GAPDH (Goodhere Biotechnology, Hangzhou, China).

### Human cervical cancer tissues and cell culture

We acquired 133 cases of cervical cancer (CC) and 47 cases of normal cervical tissues between January 2011 to December 2016 from Department of Pathology of the Eighth Affiliated Hospital, Sun Yat-sen University and the Affiliated Hospital of Guangdong Medical University. The diagnoses were conducted by three professional pathologists, and the study was approved by the Institutional Research Ethics Board of the Eighth Affiliated Hospital, Sun Yat-sen University. We purchased the human cervical cancer cell lines C33a, Hce1, HeLa, and SiHa from the China Center for Type Collection (CCTCC) (Wuhan, China). Cell lines were cultured in DMEM (Gibco, CA, USA) medium containing 10% fetal bovine serum (FBS, Sera Gld, Amarica), 100 U/mL of penicillin, and 100 U/mL of streptomycin. All of the cells were incubated in a humidified incubator in 5% CO_2_ in compressed air at 37 °C.

### Immunohistochemistry

Paraffin blocks were cut into 4-μm sections and treated routinely following the reagent instructions. After microwaving in citrate buffer for 5 min, the slides were incubated with anti-CD36 at room temperature. The sections were then incubated with a secondary antibody (MaximBio Company, Fuzhou, China), labeling was monitored using diaminobenzidine (Maxim-Bio Company, Fuzhou, China), and hematoxylin was used to stain the sections. We scored expression in accordance with the intensity (0, no staining; 1, weak staining; 2, moderate staining; 3, strong staining), and the percentage of cervical cancer cells that were stained (0, none stained; 1, < 10% stained; 2, 10–50% stained; 3, > 50% stained; 4, > 75% of all of the cervical cancer cells stained). If the product of multiplying staining intensity by the percentage of positively stained cervical cancer cells was ≥ 2, it was regarded as positive (+).

### Transfection with small interfering RNA (siRNA)

Homo sapiens CD36 siRNA was obtained from Guangzhou RiboBio Biological Technology (Guangzhou, China), and it targeted the sequence 5′-ACGTATAAGGACCTCTTTG-3′. HeLa and SiHa cells were seeded at 2 × 10^5^ cells/well in six-well plates. We transfected HeLa and SiHa cells with CD36 siRNA or control siRNA (sense strand, 5′- UUCUCCGAACGUGUCACGU TT-3′; antisense, 5′-ACGUGACACG UUCGGAGAATT-3′) with Lipo3000 at a final concentration of 100 nM, and incubated the cells at room temperature for 15 min. The complex was then added to the culture medium for subsequent experiments.

### Plasmid construction and transfection

pIRES2-ZsGreen1-CD36 was constructed and amplified by Hanbio Biotechnology (Shanghai China). We selected the cell line with G418 (600 µg/mL) for 3 weeks and expanded it, and C33a cells that overexpressed CD36 were labeled “C33a–CD36” for our study.

### Wound healing assay and trans-well assay

The C33a/CD36, C33a/vector (control), SiHa/siRNA, SiHa/nc-siRNA (control), HeLa/siRNA, and HeLa/nc-siRNA (control) cells were seeded into 12-well plates at a density of 1 × 10^5^ cells/well. The cells were then scraped with a 200-μL sterile pipette tip when they formed monolayers. After washing the cells three times with PBS, we used serum-free medium for culture, and photographed the cells at 0 and 48 h.

We performed the invasion assay using transwell plates (Costar, USA). The C33a/CD36, C33a/vector, SiHa/siRNA, SiHa/nc-siRNA, HeLa/siRNA, and HeLa/nc-siRNA cells (each at a density of 1 × 10^5^ cells/well) were added to the upper chamber with 0.2 mL of serum-free RPMI-1640; we added 0.5 mL of 10% FBS medium to the lower chamber. The cells were allowed to invade for 48 h at 37 °C. After removing the cells on the upper surface of the membrane, we stained cells on the lower aspect with trypan blue.

### Colony formation assay and evaluation of cellular apoptosis by flow cytometry

We adjusted the concentrations of C33a/CD36, C33a/vector, SiHa/siRNA, SiHa/nc-siRNA, HeLa/siRNA, and HeLa/nc-siRNA cells to appropriate densities, and then inoculated each culture dish with 200 cells at 37 °C, changing the medium every 4 days. After 2 weeks, cells were stained with trypan blue, and numbers of cell colonies were counted using a light microscope.

Analysis of cellular apoptosis was conducted strictly following the instructions of the apoptosis kit (KeyGEN BioTECH, Nanjing, China). C33a/CD36, C33a/vector, SiHa/siRNA, SiHa/nc-siRNA, HeLa/siRNA, and HeLa/nc- siRNA cells were incubated with the DNA-binding dye propidium iodide (50 ug/mL) and RNase (1.0 mg/mL) for 20 min at 37 °C in the dark. We then washed the cells and analyzed the emitted red fluorescence with a flow cytometer (BD, Heidelberg, Germany).

### Immunofluorescence

C33a/CD36, C33a/vector, SiHa/siRNA, SiHa/nc-siRNA, HeLa/siRNA, and HeLa/nc-siRNA cells were cultured in 24-well plates (at 1 × 10^5^/mL) overnight. The cells were washed twice with ice-cold PBS, fixed with 4% paraformaldehyde for 20 min, and then permeabilized with 0.5% Triton X-100 for 10 min at room temperature. The samples were then incubated with primary antibodies, including CD36 (1:50 dilution) and vimentin (1:100 dilution) at 4 °C overnight. After washing 3 times with PBS, we incubated the samples with secondary antibodies (Alexa Fluor 488, Alexa Fluor 594, 1:500 dilution) mixed with DAPI (1:1000 dilution) for 1 h in the dark. After washing three times and covering the samples with anti-fluorescence quencher, we recorded the images using a fluorescence microscope.

### Western immunoblotting analysis

We lysed the cells in a lysis buffer after washing twice with ice-cold PBS, and quantified total protein concentrations with a BCA kit (Beyotime Biotechnology, Guangzhou, China). Twenty micrograms of total protein was boiled for 5 min before being loaded onto 10% polyacrylamide gels and transferred to a polyvinylidene fluoride (PVDF) membrane. The membranes were incubated with primary antibody, including anti-CD36 (1:200 dilution), anti-E-cadherin (1:500 dilution), anti-TGF-β (1:500 dilution), anti-vimentin (1:500 dilution), anti-snail (1:500 dilution), anti-slug (1:500 dilution), anti-twist (1:500 dilution), anti-GAPDH (1:1000 dilution), and anti-β-actin (1:1000 dilution) at 37 °C overnight. Next, the membranes were incubated with a secondary antibody for 1 h, and the specific protein bands on the membranes were detected using an enhanced chemiluminescence kit (Beyotime, China).

### In vivo experiments

A nude mouse xenograft model was established using 4-week-old female BALB/C nude mice obtained from Hunan SJA Laboratory Animal Center. C33a/CD36 and C33a/vector cells (2 × 10^6^/mL) were subcutaneously injected into the lower abdomen or tail vein of the nude mice, and the tumor diameters for each mouse were measured weekly. After 5 weeks, we euthanized the mice using anesthesia, and the tumors were removed and measured. All of the animal protocols were conducted in accordance with the Institutional Animal Ethics Care Committee.

### Statistical analysis

All statistical analyses were conducted using SPSS 17.0 (SPSS, Inc., Chicago, IL, USA). We performed experiments in triplicate, and data are presented as mean ± SEM. The χ2 test was used to analyze the relationship between CD36 levels and clinicopathologic characteristics. Data from two groups were analyzed by unpaired *t* tests; and, if more than two groups, by one-way ANOVA. A *P* value of < 0.05 was considered statistically significant.

## Results

### Clinical significance of CD36 expression in cervical cancer

**Fig. 1:**
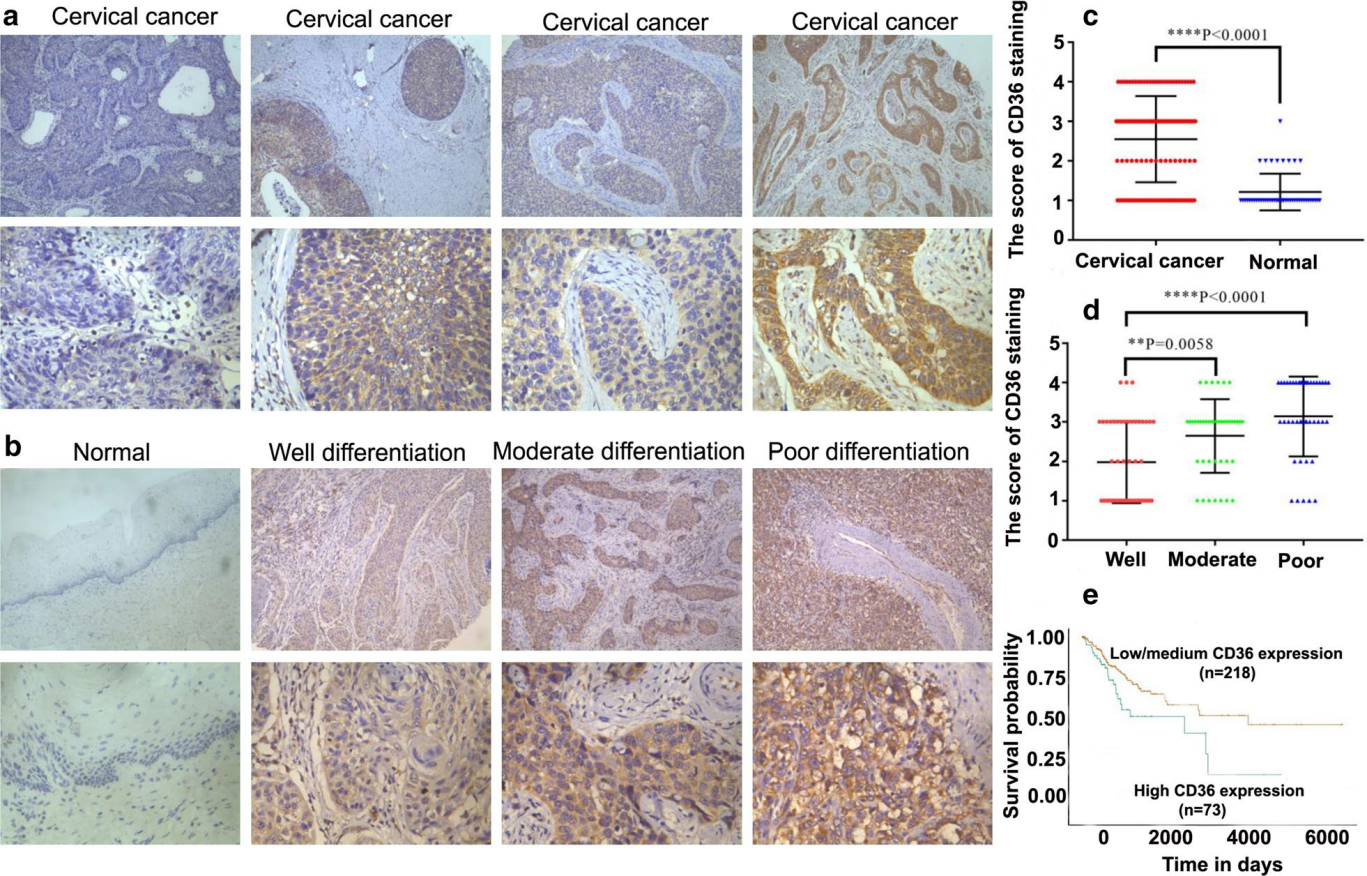
CD36 expression and its clinical significance in cervical cancer patients. **a** Over-expression of CD36 in cervical cancer (magnification: upper row, ×100 ; lower row, ×200). **b** Expression levels of CD36 in normal cervical tissues and cervical cancer with well, moderate and poor differentiation (magnification: upper row, ×100; lower row, ×200). **c** Scores for CD36 staining in cervical cancer and normal cervical tissues. **d** Scores for CD36 staining in cases of well, moderate, and poor differentiated cervical cancer. **e** Kaplan–Meier survival analysis of cervical cancer patients according to their CD36 protein expression status

After investigating the expression levels of CD36 in 133 cases of cervical cancer tissues and 47 cases of normal cervical tissues by immunohistochemistry, we found CD36 expression to be primarily located on the cell membrane or in the cytoplasm (Fig. 1a–d). As shown in Table 1, CD36 immunoreactivity was detected in 73.68% (98/133) of cervical cancer cases, whereas CD36 was only expressed in 19.15% (9/47) of normal cervical tissues. Furthermore, when we evaluated the correlation between CD36 expression and various clinicopathologic parameters of patients, we observed that CD36 expression was correlated with tumor differentiation and lymph node metastasis (*P *< 0.05, Table 2), but not with other clinicopathologic features, such as age and clinical stage. In addition, using Kaplan–Meier survival analysis and TCGA datasets, we showed that patients with cervical cancer and high CD36 expression levels had an unfavorable prognosis relative to those with low or medium CD36 expression (Fig. 1e) (*P* = 0.02). These results indicated that CD36 might be involved in the progression of cervical cancer.

**Table 1:** The expression of CD36 in cervical cancer and normal tissues

Group	N (100%)	Negative	Positive	χ^2^	P
Normal tissues	47	38	9	42.842	0.000
Cervical cancer	133	35	98

**Table 2:** Relationship between CD36 expression and clinicopathology in cervical cancer

Group	CD36 expression				
	n	Positive	Negative	χ^2^	*P*
Age					
≥ 50	66	51 (77.3%)	15 (22.7%)	0.870	0.351
< 50	67	47 (70.1%)	20 (29.9%)		
Differentiation					
Good	48	25 (52.1%)	23 (47.9%)	18.351	0.000*
Moderate	42	35 (83.3%)	7 (16.7%)		
Poor	43	38 (88.4%)	5 (11.6%)		
Clinical stage					
I	87	66 (75.9%)	21 (24.1%)	0.615	0.433
II–IV	46	32 (69.6%)	14 (30.4%)		
Lymph node metastasis					
Positive	79	64 (81.0%)	15 (19.0%)	5.389	0.020*
Negative	54	34 (63.0%)	20 (37.0%)		

* *P *< 0.05

### CD36 promotes cervical cancer cell proliferation, migration, and invasion, and inhibits apoptosis

**Fig. 2:**
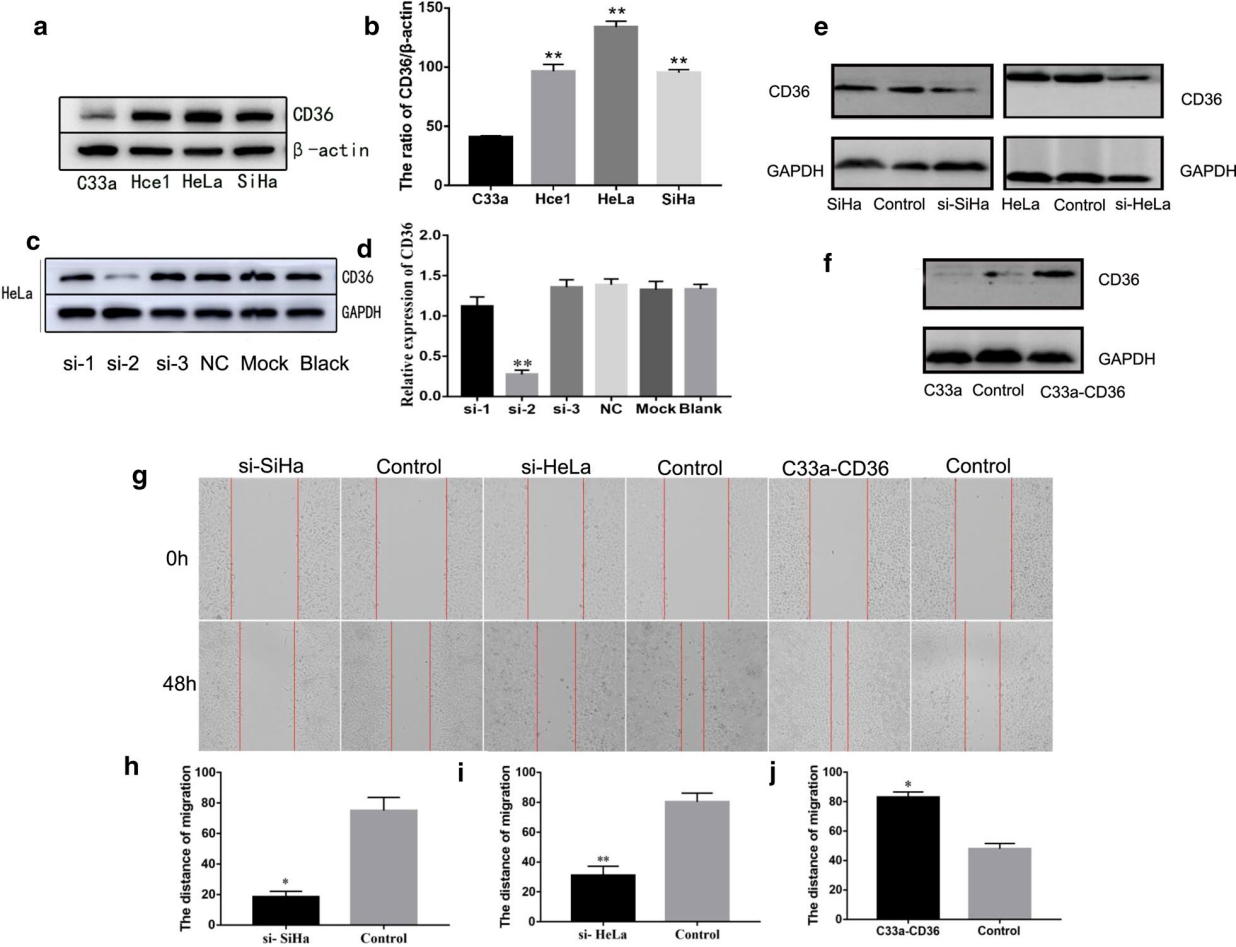
Effects of CD36 knockdown and overexpression on migration of cervical cancer cells in vitro. **a**, **b** Expression levels of CD36 in cervical cancer cell lines (**P < 0.01 vs. controls). **c**, **d** Three si-RNAs were selected to knockdown CD36 expression. Si-RNA-2 (si-2) was the most effective target sequence in our assay. **e** Expression levels of CD36 were significantly decreased by si-RNA (si-2) in SiHa and HeLa cells. **f** Expression level of CD36 was significantly enhanced after transfected with plasmid overexpressing CD36 in C33a cells. **g**–**i** Interference of CD36 expression inhibited migration both in SiHa and HeLa cells (*P < 0.05 vs. controls; **P < 0.01 vs. controls). **g**, **j** Enhancement of CD36 expression promoted migration of C33a cells (*P < 0.05 vs. controls)

To further investigate the effects of CD36 on a series of biologic processes in cervical cancer cell lines, we used Western blotting analysis to detect the expression of CD36 in C33a, Hce1, HeLa, and SiHa cells. Our data suggested that the expression of CD36 in Hce1 (96.60 ± 5.66), HeLa (134.24 ± 4.67), and SiHa (95.49 ± 2.49) cells was markedly higher than in C33a (44.77 ± 1.35) cells (Fig. 2a, b). Hence, we selected C33a, HeLa, and SiHa cells to study whether CD36 exerted tumor-promoting effects by assaying for cell migration, invasion, proliferation, and apoptosis. Cells were stably transfected with plasmids overexpressing CD36 or knocking down CD36 expression: C33a–CD36, C33a/vector (control); SiHa/siRNA, SiHa/nc-siRNA (control); and HeLa/siRNA, HeLa/nc- siRNA (control).

**Fig. 3:**
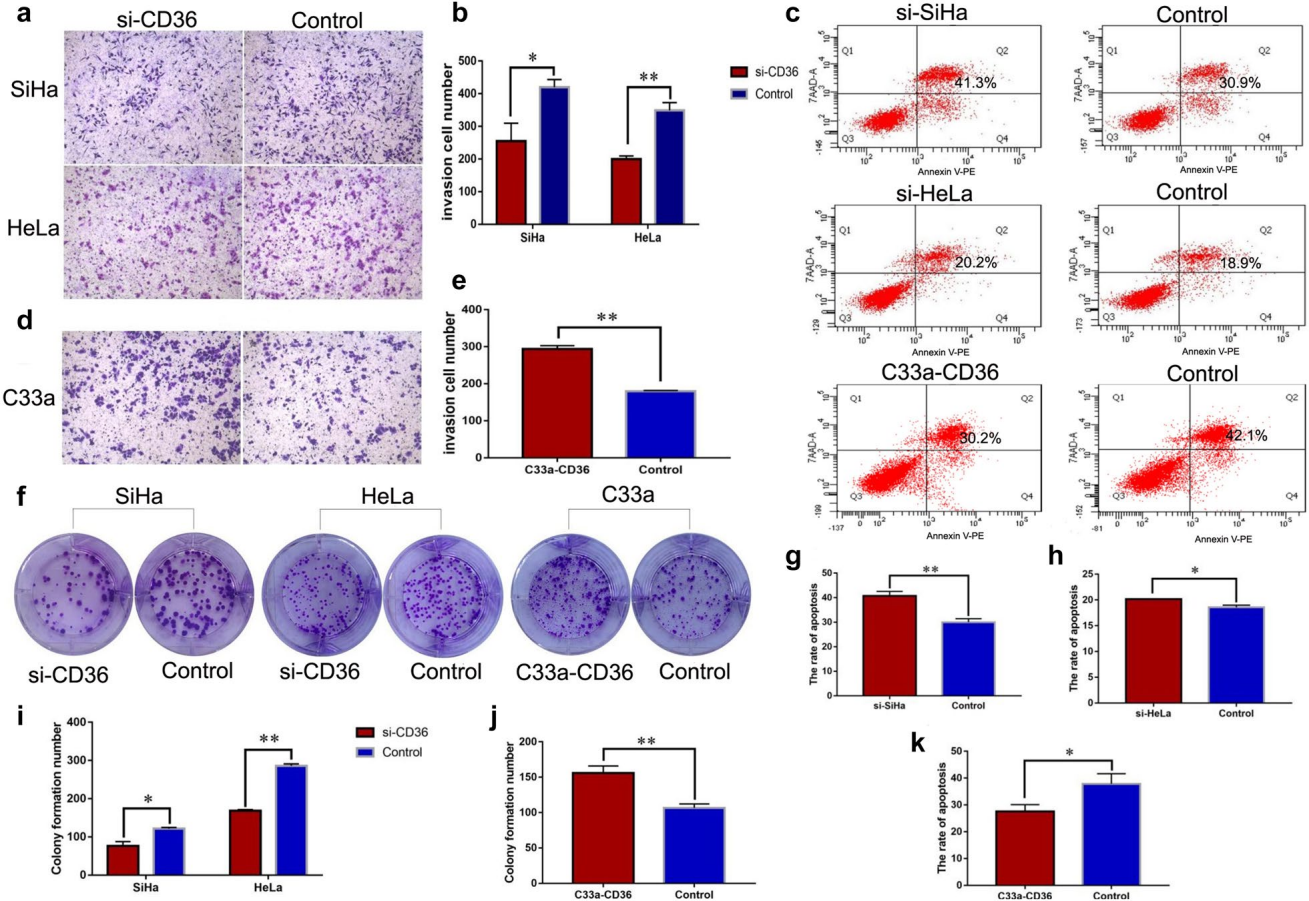
The biologic effects of CD36 on cervical cancer cell lines. **a**, **b** Knockdown CD36 expression inhibited invasion both in SiHa and HeLa cells (*P < 0.05 vs. controls; **P < 0.01 vs. controls). **d**, **e** Enhancement CD36 expression promoted invasion of C33a cells (**P < 0.01 vs. controls). **c**, **g**, **h** Knockdown CD36 expression increased the rate of apoptosis in SiHa and HeLa cells. **c**, **k** Enhancing CD36 expression inhibited the apoptotic rate of C33a cells (*P < 0.05 vs. controls). **f**, **i** interference of CD36 expression decreased colony formation in SiHa and HeLa cells (*P < 0.05 vs. controls; **P < 0.01 vs. controls). **f**, **j** Overexpression of CD36 facilitated colony formation in C33a cells (**P < 0.01 vs. controls)

As shown in Fig. 2c, d, si-RNA-2 was the most effective si-RNA sequence for interference CD36 expression. After transfected with CD36 siRNA-2 in SiHa and HeLa cells, expression levels of CD36 were significantly decreased (Fig. 2e). However, expression levels of CD36 were significantly enhanced after transfected with plasmid overexpressing CD36 in C33a cells (Fig. 2f). SiHa- and HeLa cells-transfected CD36 siRNA-2 were used to ascertain the effects of CD36 on migration and invasion of cervical cancer cells by trans-well and wound-healing assays. Compared with cells transfected with control siRNA (nc-siRNA), interference of CD36 expression by siRNA inhibited migration both in SiHa and HeLa cells (Fig. 2g–i). Conversely, enhancement of CD36 expression by transfecting a CD36 expression vector promoted migration of C33a cells (Fig. 2g, j). Similarly, overexpression of CD36 promoted invasive ability of C33a cells, while interference of CD36 expression markedly inhibited the invasion-promoting effect in SiHa and HeLa cells (Fig. 3a, b, d, e). Therefore, our evidence verified a vital role for CD36 in the promotion of cervical cancer cell migration and invasion in vitro.

Colony formation assays and flow cytometry were used to examine the proliferative and apoptotic effects of CD36 in cervical cancer cell lines. Our results revealed that high CD36 expression facilitated colony formation in C33a cells (Fig. 3f, j), while interference of CD36 expression attenuated colony formation in SiHa and HeLa cells (Fig. 3f, i). Knocking-down the expression of CD36 increased the rate of apoptosis of SiHa and HeLa cells (Fig. 3c, g, h). In contrast, enhancing CD36 expression inhibited apoptosis in C33a cells (Fig. 3c, k).

In summary, the above results suggest that CD36 plays critical roles in the biologic behavior of cervical cancer cells, promoting proliferation, migration, and invasion, but inhibiting apoptosis.

### CD36 promotes EMT in cervical cancer

**Fig. 4:**
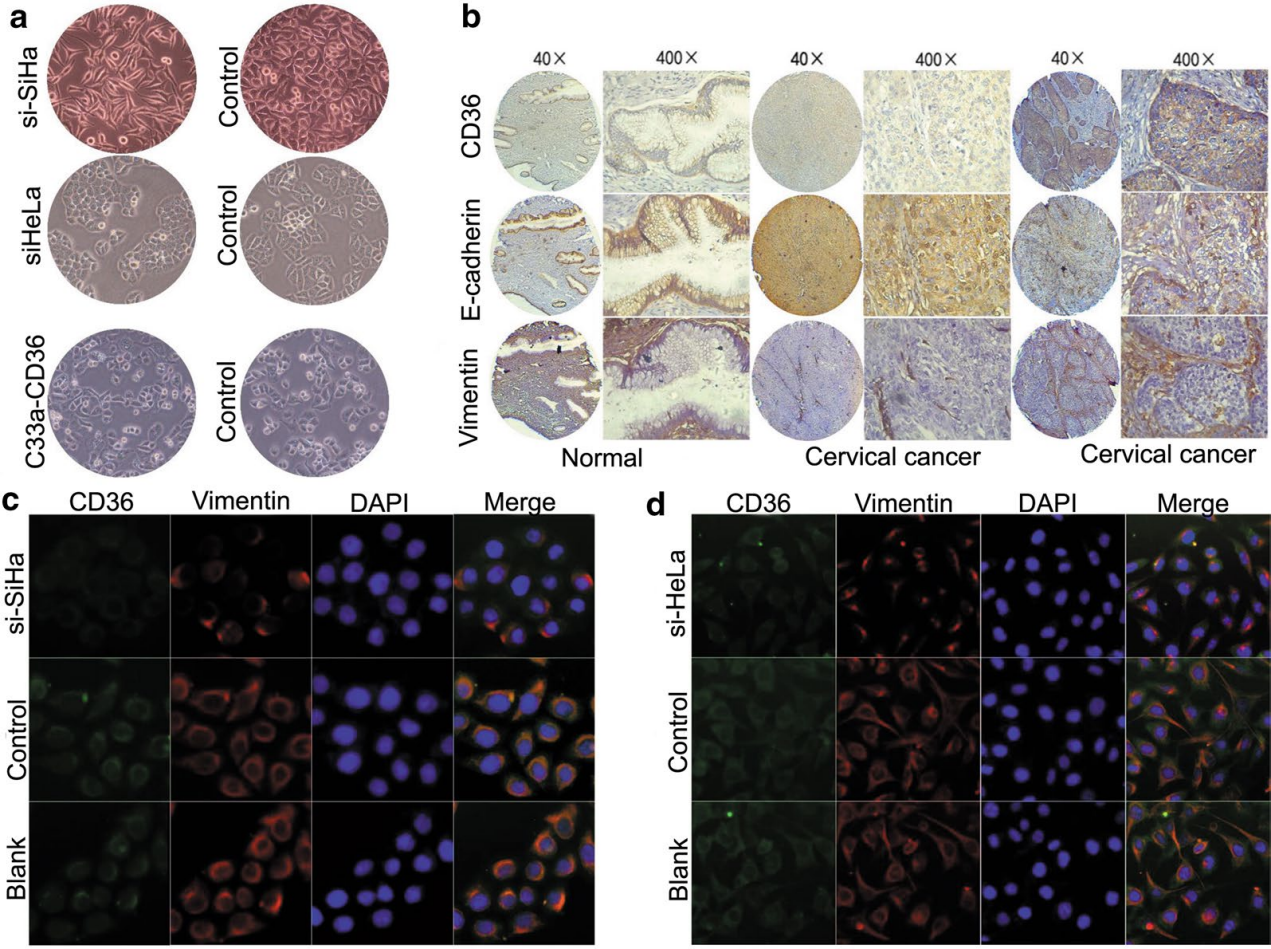
Correlation between CD36 and EMT markers using immunohistochemistry and immunofluorescence in cervical cancer cells. **a** Change in morphology of cervical cancer cells by controlling CD36 expression. **b** Correlation between CD36 and E-cadherin, vimentin. **c**, **d** Immunofluorescence staining indicated that knockdown of CD36 expression decreased expression of vimentin

We found that the morphology of SiHa and HeLa cells (normally manifesting a spindle-invasive phenotype) was replaced by a polygonal phenotype after knockdown of CD36 expression. However, the morphology of C33a cells changed from a polygonal-shaped phenotype to mesenchymal-like morphology under CD36 expression (Fig. 4a).

Having uncovered a close association between CD36 and invasion of cervical cancer cells, we sought to elucidate the effect of CD36 on the EMT in these cells. To evaluate this issue, we compared expression levels of some critical EMT markers with CD36 in 60 samples. As shown in Fig. 4b, the expression of E-cadherin was attenuated with elevated CD36 expression (*P *< 0.05), while vimentin expression levels were positively correlated with CD36 (*P *< 0.01).

We used immunofluorescence to detect the expression levels of vimentin in SiHa and HeLa cells after treatment, and demonstrated that knockdown of CD36 expression by transfection with siRNA led to diminished expression of vimentin (Fig. 4c, d). Collectively, our data suggest that CD36 participates in the EMT in cervical cancer.

### CD36 is essential for tumor growth and metastasis in vivo

**Fig. 5:**
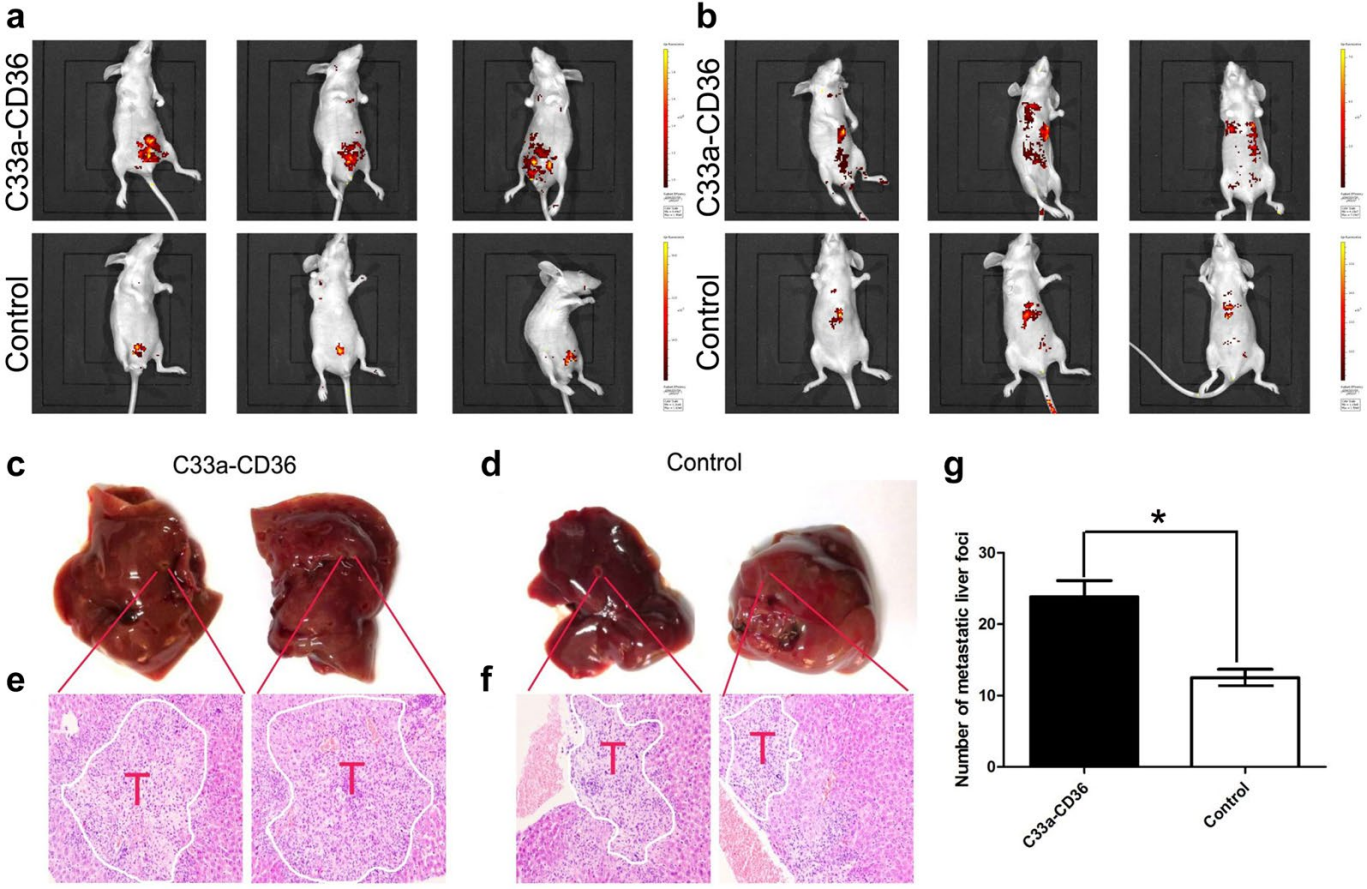
The effects of CD36 on tumor growth and metastasis in vivo. **a** CD36 over- expression promoted tumor growth in vivo. **b** Overexpression of CD36 facilitated tumor metastasis in vivo. **c**–**f** A greater number of enlarged metastatic liver foci can be observed after overexpression of CD36 (T: Tumor). **g** Statistical graph showed more numerous metastatic liver foci were observed after overexpression of CD36 (*P < 0.05)

To study the effects of CD36 on tumor metastasis in vivo, C33a–CD36 and C33a cells were injected into nude mice via the abdomen or tail vein. After 5 weeks (consistent with in vitro data), we observed that overexpression of CD36 significantly promoted tumor growth in female BALB/c-nu mice (Fig. 5a). In addition, although all of the mice that received tail vein injections of C33a–CD36 and C33a cells developed micro-metastases in the liver, more numerous and enlarged metastatic liver foci were observed after overexpression of CD36 (Fig. 5b–g). These results suggest that CD36 promotes the growth and metastasis of cervical cancer cells in vivo.

### CD36 correlates with TGF-β-mediated EMT in cervical cancer

**Fig. 6:**
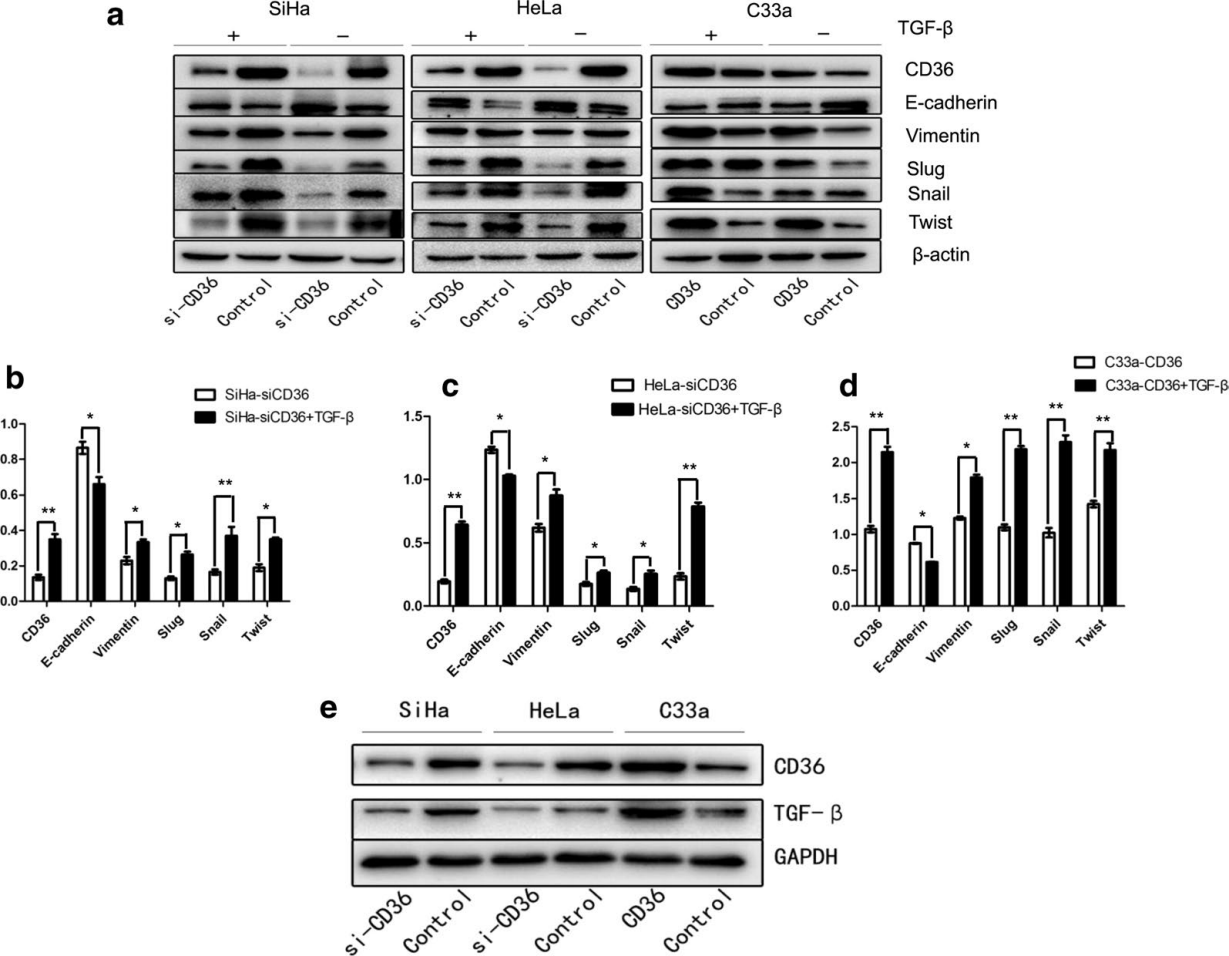
Western blots were used to analyze the association between CD36 and TGF-β-mediated EMT**. a** Knockdown of CD36 led to a dramatic increase in E-cadherin and decrease in vimentin, slug, snail, and twist both in SiHa and HeLa cells. Conversely, after transfected with exogenous CD36, these phenotypes were reversed. TGF-β treatment attenuated E-cadherin expression and enhanced the expression levels of CD36, vimentin, slug, snail, and twist in si-SiHa, si-HeLa, and C33a–CD36 cells. TGF-β regulated EMT markers though CD36. **b**–**d** Under the condition with or without TGF-β, expression levels of CD36, E-cadherin, vimentin, slug, snail, and twist in si-SiHa, si-HeLa, and C33a–CD36 cells (*P < 0.05, **P < 0.01). **e** CD36 also regulated expression levels of TGF-β

To further validate CD36 promotion of cervical cancer metastasis by regulating EMT, we attempted to detect the effect of CD36 on some critical EMT markers. As shown in Fig. 6a–d, compared with their respective control cells, knockdown of CD36 led to a dramatic increase in E-cadherin protein expression that resulted in a statistically significant decrease in vimentin, slug, snail, and twist protein expression, both in SiHa and HeLa cells. Conversely, the protein expression levels for vimentin, slug, snail, and twist were upregulated when transfected with exogenous CD36 in C33a cells, although E-cadherin was down-regulated (Fig. 6a–d).

Investigators have recently demonstrated that TGF-β is a vital inducer of EMT. Hence, we further evaluated the association among CD36, TGF-β, and EMT. In the present study we observed that after TGF-β treatment, the expression of CD36 was elevated not only in si-SiHa and si-HeLa cells, but also in C33a–CD36 cells, indicating that TGF-β promoted CD36 expression (Fig. 6a–d). More importantly, we demonstrated that TGF-β treatment attenuated E-cadherin expression and enhanced the expression levels of vimentin, slug, snail, and twist in si-SiHa, si-HeLa, and C33a–CD36 cells (Fig. 6a–d), suggesting that TGF-β synergized with CD36 on EMT via active CD36 expression. We also observed that the expression levels of TGF-β in si-SiHa cells and si-HeLa cells were down-regulated, whereas the expression levels of TGF-β were up-regulated in C33a–CD36 cells (Fig. 6e). These results imply that CD36 and TGF-β interact with each other to promote EMT in cervical cancer.

## Discussion

CD36 in its role as a cell surface receptor has been shown to promote the initiation and progression of oral carcinoma by mediating the uptake of exogenous fatty acids across the plasma membrane [13]. The investigators demonstrated that tumor metastasis in mice was significantly reduced, and that primary tumors atrophied or even disappeared, after blocking CD36 receptors [13]. These amazing research findings highlight the critical role of CD36 in promoting tumor metastasis.

In this study, we revealed that CD36 immunoreactivity in cervical cancer tissues is significantly higher than that in normal tissues. It is notable that CD36 has negative staining in 35 cervical cancer samples. These results may be explained partly by poor fixation of cervical cancer tissues. Our results presents the first clinical study showing a significant correlation between high CD36 expression and poor overall survival of cervical cancer patients. Moreover, we demonstrated that CD36 facilitated invasion and metastasis of cervical cancer cells, and this constituted a possible mechanism by which CD36 promoted EMT. More importantly, CD36 promoted the EMT process at least partially via the TGF-β signaling pathway, thus contributing to the progression of cervical cancer.

Our finding of CD36 high expression in samples with lymph node metastasis is commensurate with a recent report showing that CD36 overexpression characterized head and neck cancer stem cells that drive metastasis [13]. In addition, CD36^+^ cancer stem cells, which have multiple functions in promoting cancer progression, have been correlated with cancer initiation, chemotherapy resistance, and self-renewal activity [5, 17]. In our study, CD36 overexpression in C33a cells facilitated proliferation and invasion in vitro and aggravated tumor metastasis in a xenograft mouse model. Conversely, knockdown of CD36 expression reversed the malignant phenotype of SiHa and HeLa cells. These findings provide experimental evidence to explain the clinical observations that cervical cancer patients with high CD36 expression in tissues have higher lymph node metastasis possibilities and shorter overall survival. Thus, the potential metastasis-promoting mechanisms underlying CD36 effects, other than fatty acid uptake, must be further clarified.

EMT, a vital process promoting tumor metastasis, involves loss of the epithelial phenotype and gain of a mesenchymal phenotype [18–20]. By cooperation with Ras, TGF-β plays vital role in oncogenic EMT associated with cancer progression [21]. Similarly, Wnt [22], hedgehog [23, 24], and NF-κB [25, 26] have also been implicated in the critical pathways subserving the EMT process. Interestingly, CD36 also modulates cell-to-extracellular matrix attachment and TGF-β activation [27]. In the present study, we found that enhanced CD36 expression markedly decreased the expression level of E-cadherin (epithelial marker) and increased the expression levels of vimentin, slug, snail, and twist (mesenchymal markers) in C33a cells. Furthermore, suppression of CD36 expression reversed the expression levels of these EMT markers in SiHa and HeLa cells. More importantly, we found that TGF-β synergized with CD36 on the EMT via active CD36 expression. In addition, the expression level of TGF-β increased after exogenous transfection of CD36 in C33a cells. Therefore, we herein suggest that CD36 and TGF-β interact with each other to promote EMT in cervical cancer.

## Conclusions

In summary, our results reveal a close link between CD36 expression and the progression of cervical cancer. Moreover, CD36 promoted EMT progression at least partially by interacting with TGF-β. In future studies, we must further clarify whether CD36 regulates TGF-β-mediated EMT through the TGF-β-Smad pathway. We postulate that after validation in large clinical cohorts, CD36 will be an effective target for guiding individualized clinical therapy of cervical cancer.

## Data Availability

All data generated or analyzed during this study are included in this article.

## Abbreviations

HPV: human papillomavirus
EMT: epithelial–mesenchymal transition
siRNA: small interfering RNA
TSP-1: thrombospondin-1
PVDF: polyvinylidene fluoride
SP: streptavidin-perosidase
DAB: diaminobenzidine
CCTCC: China Center for Type Collection
FBS: fetal bovine serum
TGF-β: transforming growth factor-β

## References

[1] Ma C, Zhang Y, Li R (2018). Risk of parametrial invasion in women with early stage cervical cancer: a meta-analysis. Arch Gynecol Obstet.

[2] Shu L, Zhang Z, Cai Y (2018). MicroRNA-204 inhibits cell migration and invasion in human cervical cancer by regulating transcription factor 12. Oncol Lett..

[3] Liu Y, Yang Y, Li L (2018). LncRNASNHG1 enhances cell proliferation, migration, and invasion in cervical cancer. Biochem Cell Biol.

[4] Zhao J, Zhi Z, Wang C (2017). Exogenous lipids promote the growth of breast cancer cells via CD36. Oncol Rep.

[5] Hale JS, Otvos B, Sinyuk M (2014). Cancer stem cell-specific scavenger receptor CD36 drives glioblastoma progression. Stem Cells.

[6] Olonisakin TF, Li H, Xiong Z (2016). CD36 provides host protection against *Klebsiella pneumoniae* intrapulmonary infection by enhancing lipopolysaccharide responsiveness and macrophage phagocytosis. J Infect Dis.

[7] Mwaikambo BR, Sennlaub F, Ong H (2006). Activation of CD36 inhibits and induces regression of inflammatory corneal neovascularization. Invest Ophthalmol Vis Sci.

[8] Huangfu N, Xu Z, Zheng W (2018). LncRNA MALAT1 regulates oxLDL-induced CD36 expression via activating β-catenin. Biochem Biophys Res Commun.

[9] Choromańska B, Myśliwiec P, Choromańska K (2017). The role of CD36 receptor in the pathogenesis of atherosclerosis. Adv Clin Exp Med..

[10] Holmes RS (2012). Comparative studies of vertebrate platelet glycoprotein 4 (CD36). Biomolecules..

[11] DeFilippis RA, Chang H, Dumont N (2012). CD36 repression activates a multicellular stromal program shared by high mammographic density and tumor tissues. Cancer Discov.

[12] Kennedy DJ, Kashyap SR (2011). Pathogenic role of scavenger receptor CD36 in the metabolic syndrome and diabetes. Metab Syndr Relat Disord..

[13] Pascual G, Avgustinova A, Mejetta S (2017). Targeting metastasis-initiating cells through the fatty acid receptor CD36. Nature.

[14] Goto M, Osada S, Imagawa M (2017). FAD104, a regulator of adipogenesis, is a novel suppressor of TGF-β mediated EMT in cervical cancer cells. Sci Rep..

[15] Nath A, Li I, Roberts LR (2015). Elevated free fatty acid uptake via CD36 promotes epithelial–mesenchymal transition in hepatocellular carcinoma. Sci Rep..

[16] Hou Y, Wu M, Wei J (2015). CD36 is involved in high glucose-induced epithelial to mesenchymal transition in renal tubular epithelial cells. Biochem Biophys Res Commun.

[17] Ye H, Adane B, Khan N (2016). Leukemic stem cells evade chemotherapy by metabolic adaptation to an adipose tissue niche. Cell Stem Cell.

[18] Tanaka T, Goto K, Iino M (2017). Sec8 modulates TGF-beta induced EMT by controlling N-cadherin via regulation of Smad3/4. Cell Signal.

[19] Lin Y, Mallen-St Clair J, Wang G (2016). p38 MAPK mediates epithelial–mesenchymal transition by regulating p38IP and Snail in head and neck squamous cell carcinoma. Oral Oncol.

[20] Xu T, Zhou M, Peng L (2014). Upregulation of CD147 promotes cell invasion, epithelial-to-mesenchymal transition and activates MAPK/ERK signaling pathway in colorectal cancer. Int J Clin Exp Pathol..

[21] Zavadil J, Böttinger EP (2005). TGF-beta and epithelial-to-mesenchymal transitions. Oncogene.

[22] Li Y, Hively WP, Varmus HE (2000). Use of MMTV-Wnt-1 transgenic mice for studying the genetic basis of breast cancer. Oncogene.

[23] Wolf I, Bose S, Williamson EA (2007). FOXA1: growth inhibitor and a favorable prognostic factor in human breast cancer. Int J Cancer.

[24] Li X, Deng W, Nail CD (2006). Snail induction is an early response to Gli1 that determines the efficiency of epithelial transformation. Oncogene.

[25] Huber MA, Kraut N, Beug H (2005). Molecular requirements for epithelial–mesenchymal transition during tumor progression. Curr Opin Cell Biol.

[26] Huber MA, Azoitei N, Baumann B (2004). NF-kappaB is essential for epithelial–mesenchymal transition and metastasis in a model of breast cancer progression. J Clin invest..

[27] Silverstein RL, Febbraio M (2009). CD36, a scavenger receptor involved in immunity, metabolism, angiogenesis, and behavior. Sci Signal..

